# Screening Snake Venoms for Toxicity to *Tetrahymena Pyriformis* Revealed Anti-Protozoan Activity of Cobra Cytotoxins

**DOI:** 10.3390/toxins12050325

**Published:** 2020-05-15

**Authors:** Olga N. Kuleshina, Elena V. Kruykova, Elena G. Cheremnykh, Leonid V. Kozlov, Tatyana V. Andreeva, Vladislav G. Starkov, Alexey V. Osipov, Rustam H. Ziganshin, Victor I. Tsetlin, Yuri N. Utkin

**Affiliations:** 1 Gabrichevsky Research Institute of Epidemiology and Microbiology, ul. Admirala Makarova 10, Moscow 125212, Russia; filiana87@mail.ru; 2 Shemyakin-Ovchinnikov Institute of Bioorganic Chemistry, ul. Miklukho-Maklaya 16/10, Moscow 117997, Russia; evkr@mail.ru (E.V.K.); damla-sofia@yandex.ru (T.V.A.); vladislavstarkov@mail.ru (V.G.S.); osipov-av@ya.ru (A.V.O.); rustam.ziganshin@gmail.com (R.H.Z.); victortsetlin3f@gmail.com (V.I.T.); 3 Mental Health Research Centre, Kashirskoye shosse, 34, Moscow 115522, Russia; elcher10@yandex.ru

**Keywords:** snake venom, cytotoxin, protozoa, ciliate *Tetrahymena pyriformis*, immobilization, membrane rupture

## Abstract

Snake venoms possess lethal activities against different organisms, ranging from bacteria to higher vertebrates. Several venoms were shown to be active against protozoa, however, data about the anti-protozoan activity of cobra and viper venoms are very scarce. We tested the effects of venoms from several snake species on the ciliate *Tetrahymena pyriformis*. The venoms tested induced *T. pyriformis* immobilization, followed by death, the most pronounced effect being observed for cobra *Naja sumatrana* venom. The active polypeptides were isolated from this venom by a combination of gel-filtration, ion exchange and reversed-phase HPLC and analyzed by mass spectrometry. It was found that these were cytotoxins of the three-finger toxin family. The cytotoxins from several cobra species were tested and manifested toxicity for infusorians. Light microscopy revealed that, because of the cytotoxin action, the infusorians’ morphology was changed greatly, from teardrop-like to an almost spherical shape, this alteration being accompanied by a leakage of cell contents. Fluorescence microscopy showed that the fluorescently labelled cytotoxin 2 from cobra *N. oxiana* was localized mainly at the membrane of killed infusorians, indicating that cytotoxins may kill *T. pyriformis* by causing membrane rupture. This work is the first evidence of the antiprotozoal activity of cobra venom cytotoxins, as demonstrated by the example of the ciliate *T. pyriformis*.

## 1. Introduction

Snake venoms contain a pool of bioactive compounds acting on many different species. The most studied are the venom effects on the vertebrates, while the effects on invertebrates are much less investigated. As for the action on different groups of invertebrates, there are some data about snake venom toxicity to arthropods [1,2,3], the most active against cockroaches being cytotoxins from cobra venom [1]. Antibacterial properties of snake venoms were also tested [4,5] and some active proteins were identified. It was found that phospholipases A2 [6], L-amino acid oxidases [7] and cathelicidins [8] possess antimicrobial properties. There are also some data about the influence of snake venoms on protozoa [9,10]. Recently the in vivo and in vitro antileishmanial activity of krait *Bungarus caeruleus* [11] and cobra *Naja oxiana* [12] venoms was demonstrated. It was also reported that the venom of the cobra *Naja nigricollis* possessed the capacity to kill *Trypanosoma brucei*, a parasitic protozoan [13]. Furthermore, a disintegrin isolated from *Cerastes cerastes* venom exhibited antiparasitic activity on *Leishmania infantum* promastigotes [14].

Protozoa are a diverse group of single-celled eukaryotic organisms with animal-like behaviours such as motility and predation. Protozoa have been divided traditionally on the basis of their means of locomotion into Flagellates, Amoeboids, Sporozoans and Ciliates. Some protozoa are human parasites, causing life-threatening diseases such as malaria, toxoplasmosis, Chagas disease, leishmaniasis, and several others. Many protozoa are also causative agents of dangerous diseases in domestic and wild animals. Some diseases are shared between species, for example, toxoplasmosis and leishmaniasis affect both humans and animals, while others are specific only for animals. Thus, the ciliates *Ichthyophthirius multifiliis* and *Cryptocaryon irritans*, commonly known as ich and marine ich, respectively, are highly pathogenic for fishes. These ciliates are responsible for diseases causing significant economic losses to the global aquaculture industry [15], however options for control of diseases are extremely limited. Other ciliates from the *Tetrahymena* genus are relatives of *I. multifiliis* [16]. *Tetrahymena* spp. are extremely motile free-living organisms, but occasionally they can become parasitic and affect some fish species. Thus, *Tetrahymena* sp., also known as the guppy-killer parasite, is a main infectious agent of guppies [17]. Recently high mortality due to *Tetrahymena* sp. infection in laboratory-maintained zebrafish was reported [18].

At the same time some ciliates such as *Paramecium caudatum, Tetrachymena, Oxytricha trifallax* are model organisms used in molecular biology. The ciliated protozoa *Tetrahymena* sp. has been used in toxicology for decades as a model organism as well as for environmental research to assess the cytotoxicity of chemicals and environmental pollutants [19]. A conventional colorimetric *Tetrahymena* assay is based on the reduction of 3-(4,5-dimethyl-2-thiazol-2-yl)-2,5-diphenyl- 2*H*-tetrazolium bromide (MTT) [20]. However, the competing interaction of MTT with some other chemicals or pollutants present in samples cannot be completely excluded. Recently the method based on counting freely moving *T. pyriformis* organisms was developed, which did not require any additional chemicals [21].

In this study, using the new method we investigated the effects of snake venoms on ciliate *Tetrahymena pyriformis* and found that cobra venoms are lethal to this species, the cobra *Naja sumatrana* venom being the most active. Analysis of this venom demonstrated that the lethal effect was produced by cytotoxins of the three-finger family. Cytotoxins from other cobra venoms were also lethal to the ciliate. Thus, for the first time it was demonstrated that snake cytotoxins have antiprotozoal activity, using the example of the ciliate *T. pyriformis*.

## 2. Results

To find new polypeptides toxic to protozoa, venoms of several snake species including krait, vipers and cobras were tested for toxicity to the ciliate *T. pyriformis*. The method based on automatic registration of moving organisms was used. If ciliates are alive, they move constantly. To estimate the number of living microorganisms, the number of cells that have changed their location during a certain observation time should be counted. This concept is realized in a BioLaT-3.2 instrument that is designed for automated observation and computer-aided fixation of objects in a visual field [21]. We have used the BioLaT-3.2 for automated biotesting of venoms for toxicity to *T. pyriformis* ciliates.

**Figure 1:**
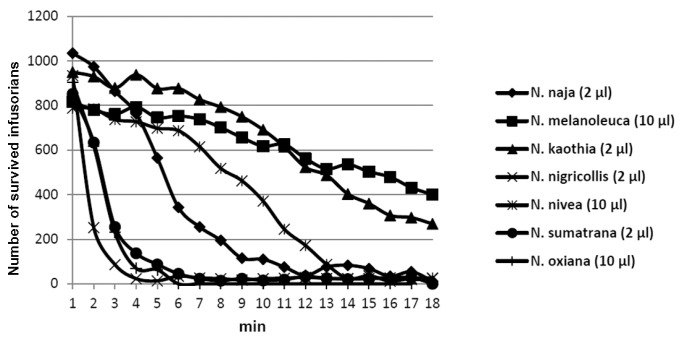
Anti-protozoan activity of cobra venoms. Indicated volume of cobra venom solution in water (50 mg/mL) was added to the instrument well containing 300 μL of infusoria suspension comprising about 800–1000 microorganisms and the number of moving (survived) organisms was counted over the time using the BioLaT-3.2 instrument.

**Figure 2:**
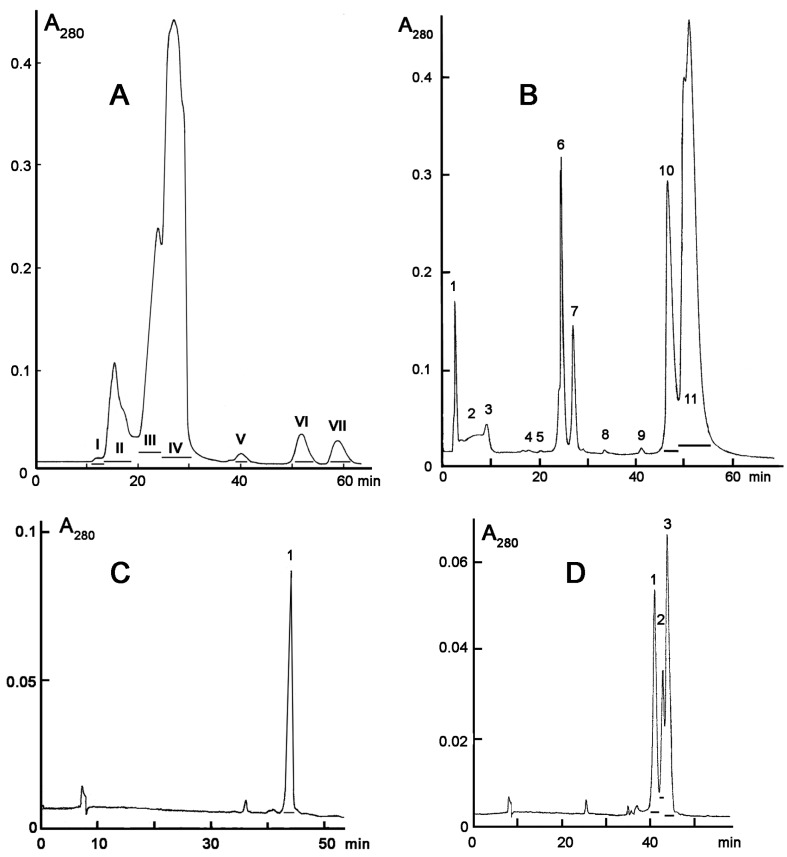
Separation of cobra *Naja sumatrana* venom by gel-filtration (**A**), ion-exchange (**B**) and reversed phase chromatography (**C**,**D**). A. Gel-filtration was performed using Superdex^TM^ 75 column equilibrated with 0.1 M ammonium acetate buffer (pH 6.2) at flow rate of 0.5 mL/min. The collected fractions are underlined. B. The toxic faction IV (Figure 2A) was separated on a HEMA BIO 1000 CM column (8 × 250 mm, Tessek, Prague, Czech Republic) applying a gradient of 5–700 mM ammonium acetate (pH 7.5) in 140 min, at a flow rate of 0.5 mL/min. Fractions obtained (Figure 2B) were screened for lethal activity and toxic fractions 10 and 11 were further separated on a Bio Wide Pore C18 column (10 × 250 mm) in a gradient of acetonitrile 20–50% in 60 min in the presence of 0.1% trifluoroacetic acid, at a flow rate of 2.0 mL/min. C – separation of fraction 10 (Figure 2B); D – separation of fraction 11 (Figure 2B). In panels B, C and D, the fractions toxic to infusorians are indicated by horizontal bars.

**Figure 3:**
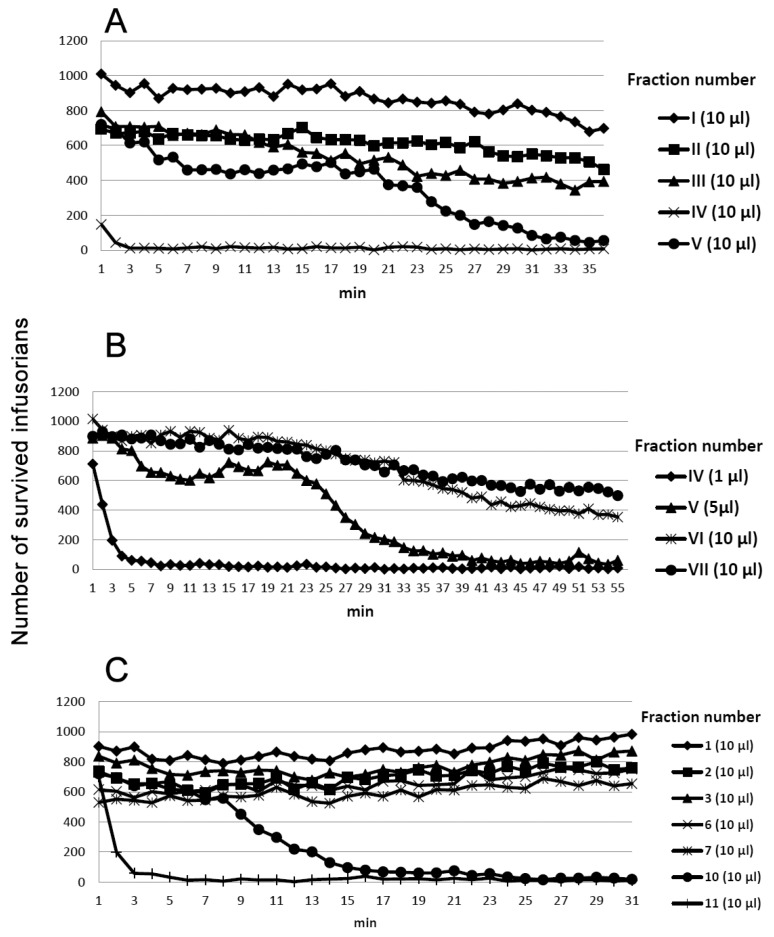
Influence of fractions obtained during separation of *N. sumatrana* venom on survival of *T. pyriformis.* (**A**,**B**) – fractions obtained after gel-filtration and numbered according to Figure 2A.; (**C**) – fractions obtained after ion-exchange chromatography and numbered according to Figure 2B. One fourth of each fraction was freeze-dried and dissolved in 100 μL of water. Each toxin solution was added to 300 μL of infusorian suspension and the number of moving (survived) microorganisms was counted using BioLaT-3.2 instrument. The volume added is indicated in brackets.

**Figure 4:**
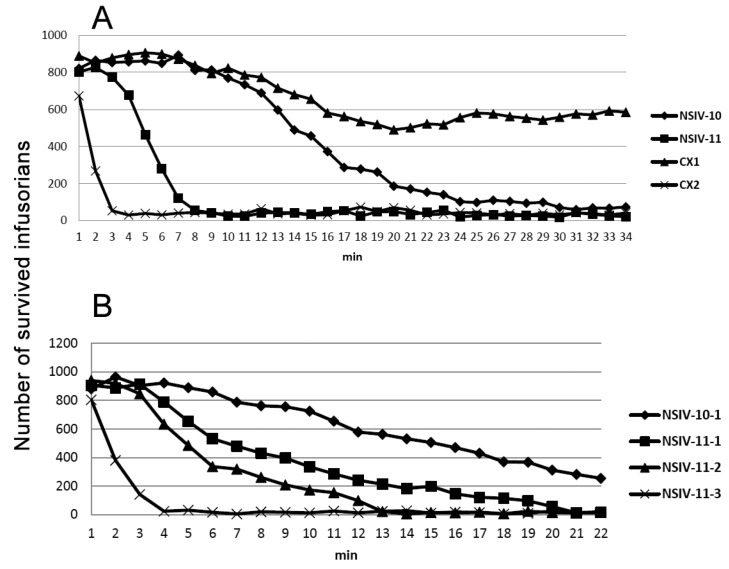
The toxicity of cytotoxins from *N. sumatrana* (NS) and *N. oxiana* (CX1 and CX2) venoms to *T. pyriformis*. Fractions and toxins were dissolved in water at the concentration of 20 mg/mL. 10 μL of each toxin solution was added to 200 μL of infusorian suspension (about 800 microorganisms) and the number of survived microorganisms was counted using the BioLaT-3.2 instrument. Final toxin concentration was 0.95 mg/mL. (**A**) NSIV-10 and NSIV-11 – fractions 10 and 11 from Figure 2B, respectively; CX1 and CX2 – cytotoxins 1 and 2 from *N. oxiana*. (**B**) NSIV-10-1 – fraction 1 from Figure 2C. NSIV-11-1, NSIV-11-2 and NSIV-11-3 – fractions 1, 2 and 3 from Figure 2D, respectively.

The venom solution was added to the instrument chamber containing infusorians and the number of moving, i.e., live microorganisms, was counted over 30–60 min. The venoms of seven cobras, five vipers, three pit vipers and one krait species were tested. We have found that only cobra venoms were lethal to *T. pyriformis* at concentrations of less than 2 mg/mL. Among the venoms of the seven cobra species (*N. kaouthia, N. melanoleuca, N. naja, N. nigricollis, N. nivea, N. oxiana, N. sumatrana)* studied (Figure 1), those of *N. nigricollis* and *N. sumatrana* were the most active (Table 1), killing infusorians at concentration of 0.33 mg/mL in less than 10 min (Figure 1). *N. sumatrana* venom killed all infusorians in 6 min and was chosen for further studies. It was fractionated using different types of liquid chromatography (Figure 2) and every fraction obtained was checked for toxicity to *T. pyriformis* (Figure 3 and Figure 4).

One fourth of each fraction was freeze-dried and dissolved in 100 μL of water. One tens of each solution, representing equal portions of the tested fractions, was used for the activity measurement. Among the fractions obtained by gel-filtration of the crude *N. sumatrana* venom, so called main toxic fraction IV (Figure 2A) was the most active against infusorians killing practically all microorganisms in less than 5 min (Figure 3A,B). A strong decrease in the number of cells at the beginning of experiment (Figure 3A, 10 µL fraction IV) is explained by very rapid cell death which occurred during the probe preparation due to the high concentration of toxins. The fraction V was less active and required more than half an hour to kill all infusorians in a counting well (Figure 3A,B).

Other fractions did not result in the death of microorganisms even after one hour of incubation. The fraction IV containing mostly cytotoxins and neurotoxins was further separated by ion-exchange chromatography (Figure 2B). The most basic fractions 10 and 11 were found to be toxic to *T. pyriformis* and killed all microorganisms within 15 and 5 min, respectively (Figure 3C). All other fractions obtained after ion exchange chromatography were not toxic at all. The fractions 10 and 11 were further fractionated by reversed-phase HPLC (Figure 2C,D), and the activity of toxins obtained was tested (Figure 4). Toxin in fraction 3 (Figure 2D), referred further as NsIV-11-3, was the most active, while others possessed lower, nevertheless pronounced toxicity to infusorians (Table 2). The toxin from fraction 1 in Figure 2C, referred further as NsIV-10-1, was also fairly toxic (Table 2).

The toxins NsIV-10-1 and NsIV-11-3 were characterized by mass spectrometry. The molecular masses determined for them were 6718 and 6758 Da, respectively. In cobra venoms, cytotoxins and short-chain α-neurotoxins possess molecular masses in this range. To identify the toxins isolated, the proteins were subjected to trypsinolysis and molecular masses of the peptides obtained were determined by MALDI mass spectrometry (Table 3). Comparison of masses determined with those of known toxins revealed the similarity of our toxins to cytotoxins from cobra *N. sputatrix* venom.

**Figure 5:**
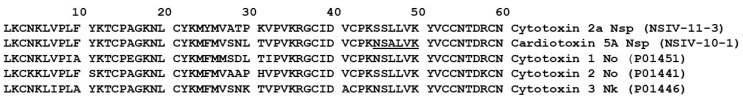
Amino acid sequences of cobra cytotoxins. Fragment 45–50 not identified by MALDI mass spectrometry in NSIV-10-1 is underlined. Nsp – *N. sputatrix*, No- *N. oxiana*, Nk – *N. kaouthia*.

According to mass spectrometry data, toxin NsIV-11-3 was identical to cytotoxin 2a from *N sputatrix* (UniProtKB accession number Q9PST4). Toxin NsIV-10-1 was very similar to cardiotoxin 5A from *N. sputatrix* (UniProtKB accession number O73857) with the exception of the fragment 45–50 for which no corresponding mass was found in the mass spectrum of toxin tryptic hydrolysate (Figure 5).

**Figure 6:**
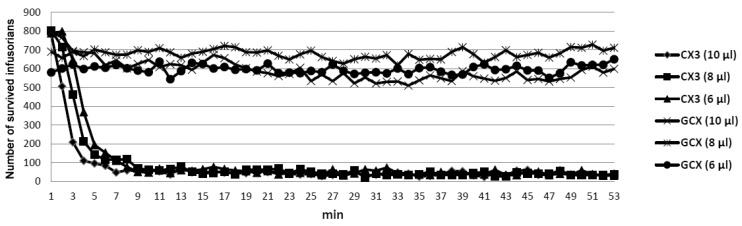
The toxicity of cytotoxin 3 (CX3) and glycosylated cytotoxin 3 (GCX) from *N. kaouthia* venom to *T. pyriformis*. The conditions for activity measurements were the same as in Figure 4.

To check if other cobra venom cytotoxins possess toxicity to *T. pyriformis*, cytotoxins 1 and 2 from *N. oxiana* as well as cytotoxin 3 from *N. kaouthia* were tested (Figure 4 and Figure 6).

All these cytotoxins were active against *T. pyriformis* (Table 2). The highest toxicity was observed for cytotoxin 2 *N. oxiana* and toxin NsIV-11-3 which killed almost all the cells in only 3–4 min at concentration of about 150 μM. Interestingly, glycosylated cytotoxin 3 *N. kaouthia*, containing carbohydrate moiety at asparagine 29 was not active in this test (Figure 6).

**Figure 7:**
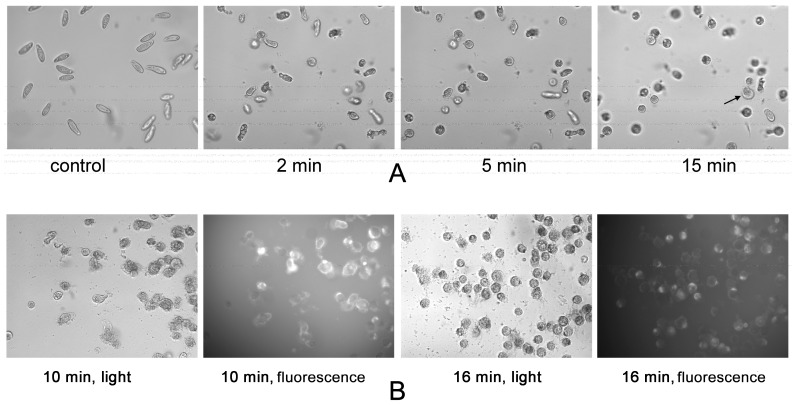
Microscopic images of cytotoxin-treated *T. pyriformis*. (**A**) Normal teardrop-like shaped *T. pyriformis* cells (control) were treated with cytotoxin 2 (1 mg/mL) during the time indicated. The cellular shape became spherical during the treatment and the leakage of cell content became evident as indicated by arrow. (**B**) *T. pyriformis* cells were incubated with fluorescently labeled cytotoxin 2 (1 mg/mL) during the time indicated. The photographs were taken in light transmission mode (light) and fluorescence mode (fluorescence).

To get information about a possible molecular mechanism of cytotoxin action on infusorians, the light and fluorescent microscopy was used. The addition of cytotoxin 2 from *N. oxiana* induced infusorian immobilization followed by strong changes in their morphology (Figure 7A). The teardrop-like form (Figure 7A, control) was transformed to almost spherical one (Figure 7A, 5 min). At a longer time (more than10 min), the leakage of cell content was observed (Figure 7A, 15 min). To localize the cytotoxin binding on the infusorians, the fluorescent analogue of cytotoxin 2 from *N. oxiana* was used. It was found that a small amount of fluorescent toxin was localized inside cells (Figure 7B, 10 min, fluorescence mode), but the main staining by toxin was observed at the membrane of dead infusorians (Figure 7B, 16 min, fluorescence mode).

## 3. Discussion

Snake venoms have evolved as a potent weapon for prey immobilization and defense from predators. They comprise complex mixtures of peptides and proteins as well as of non-proteinaceous compounds optimized to act on vital systems in the organisms of the prey or predator. Snake predators are fairly large vertebrate animals and snakes themselves feed on relatively large animals, including vertebrates and arthropods. It was assumed quite reasonable that the main toxic venom components were directed against these animals and the biological effects of venoms were studied mostly on these animals. Data about the action of snake venoms and their components on microorganisms are not so numerous [4,5]. However, some proteins with antimicrobial properties were identified. In particular, they include phospholipases A2 [6], L-amino acid oxidases [7] and cathelicidins [8]. Venoms of several snake species were shown to be toxic to protozoans [9,10,11,12] and identification of proteins manifesting anti-protozoan activity in viperid venoms revealed that phospholipase A2 [22], metalloproteinase [23], L-amino acid oxidase [24] and disintegrin [14] are toxic to different protozoa. For example, BnSP-7 toxin, a catalytically inactive phospholipase A2 from *Bothrops pauloensis* snake venom, showed activity against promastigote *Leishmania amazonensis* parasite forms, inhibiting parasite proliferation by 60–70% at toxin concentrations of 50–200 μg/mL 96 h after treatment [22]. However, the data about influence of cobra venom on protozoa were very limited. Thus, it was shown that *N. haje* venom exerted a significant growth inhibition of *Trypanosoma cruzi* and *Leishmania donovani infantum* parasites [25] and the venom of *N. oxiana* revealed remarkable anti-leishmanial effects [12].

It should be noticed that protozoa are not only human parasites, causing life threatening diseases in humans, but they are also pathogens of serious diseases in wild and domestic animals producing substantial losses in agriculture. In particular, the ciliates *Ichthyophthirius multifiliis* and *Cryptocaryon irritans*, highly pathogenic for fishes, have been mentioned above [15].

To get more information about the anti-protozoan activity of cobra venoms we have used infusorian *T. pyriformis* as a model organism. The survival of animals was monitored using an original method [21] in which the mobility of each organism was registered separately and dead unmoving organisms were not registered. Among several snake species (vipers, kraits, and cobras) studied, only cobra venoms were found to be lethal to infusorian *T. pyriformis*. The venoms of seven cobra species (*N. kaouthia, N. melanoleuca, N. naja, N. nigricollis, N. nivea, N. oxiana, N. sumatrana)* were tested (Figure 1) to choose the most active one. The highest activity was manifested by the venoms of *N. nigricollis* and *N. sumatrana*, and that of *N. sumatrana* was chosen for the further studies. To identify the active compound, this venom was fractionated by three different types of liquid chromatography (Figure 2), and the activity of each fraction obtained was determined (Figure 3 and Figure 4). As a result several toxins active agains *T. pyriformis* were isolated. The structures for the most active ones were characterized in detail by MALDI mass spectrometry, and it was shown that these are cytotoxins of the three-finger toxin family. It should be mentioned that in protein data banks there were no amino acid sequence data for toxins from *N. sumatrana*. The paper on the proteomic characterization of *N. sumatrana* venom contains only fragmentary data on the cytotoxin amino acid sequences and does not allow the toxin assignment [26]. Recently de novo venom-gland transcriptomics of *N. sumatrana* from West Malaysia was conducted using next-generation sequencing technology [27]. The transcripts encoding toxin identical to cytotoxin 2a from *N. sputatrix* were the most abundant among the all transcripts identified. According to MALDI mass spectrometry data, one of the toxins isolated in this work was identical to cytotoxin 2a and the other one was very similar to cardiotoxin 5A from *N. sputatrix* venom.

Earlier we have studied the interaction of several cytotoxins and their fluorescent analogues with artificial membranes [28] and mammalian cell lines [29]. These toxins including cytotoxins 1 and 2 from *N. oxiana* as well as cytotoxin 3 from *N. kaouthia* were tested on infusoria (Figure 4 and Figure 6). All cobra cytotoxins used in this study were toxic to the infusorians, cytotoxin 2 *N. oxiana* being the most active. The higher activity of *N. sumatrana* venom as compared with that of *N. oxiana* may be explained by higher cytotoxin content in the former. To get some information about possible interacting site in cytotoxin, we tested the activity of glycosylated cytotoxin 3 *N. kaouthia* containing carbohydrate moiety at asparagine 29. Earlier we have shown [30] that this post-translational modification resulted in decrease of the cytotoxic activity of the glycoprotein towards HL60 cells by about two orders of magnitude. This toxin was completely inactive against infusoria (Figure 6), which means that the central toxin loop containing asparagine 29 is involved in the anti-protozoan activity.

It should be mentioned that according to differences in the amino acid sequences of cobra cytotoxins within the so-called central loop II, they can be classified into two groups: P-type and S-type. P- and S-types differ by the presence of either Pro-31(30) or Ser-29(28) residues, respectively [31]. The difference in biological activity between these two types was observed pointing to the more efficient interaction of P-type cytotoxin with membrane [28,32]. The cytotoxins studied in our work belong to different types; CX2 *N. oxiana* and NSIV-11-3 are of P-type whereas CX1 *N. oxiana*, CX3 *N. kaouthia* and NSIV-10-1 are of S-type (Table 2). Based on a comparison of toxicity for ciliates, a very cautious conclusion can be drawn that the p-type exhibits higher toxicity (Table 2). However, in order to make a more rigorous conclusion, additional experiments with a large number of toxins are required.

To establish a possible mechanism of the cytotoxin action, the light and fluorescence microscopy studies were performed: they showed that, due to the cytotoxin action, the morphology of infusorians changed greatly from teardrop-like to almost spherical shape. If incubation with the cytotoxin was continued for the longer time (more than 10 min), the leakage of cell content was observed (Figure 7). Fluorescently labelled cytotoxin 2 *N. oxiana* was found to be localized mainly at the membrane of dead infusorians, however it was also visible in lesser amount inside the infusorians. These data suggest that cytotoxins kill *T. pyriformis* by membrane rupture.

As discussed in the Introduction, *Tetrahymena* spp. in some cases can become parasitic and affect certain fish species. Thus, it can seriously infect guppies and is thus called the guppy-killer parasite [17]. Moreover, *Tetrahymena* sp. infection was reported for laboratory-maintained zebrafish [18]. Interestingly, the most active cytotoxins (e.g., CX2 *N. oxiana*) kill *Tetrahymena* in less than 5 min at concentration of 1 mg/mL while LD_50_ for cytotoxin of the cobra *Naja mossambica mossambica* to fish *Aphanius dispar Ruppell* (Pisces, Cyprinodontidae) was about 0.4 mg/mL at external application to the surrounding water [33]. Lethality, based on complete fish immobility, was determined after 60 min. It is quite possible that *Tetrahymena* might be killed much faster at this toxin concentration. Thus, cytotoxins may be considered as a possible treatment for fishes infected with *Tetrahymena.* However, this suggestion needs further experimental studies.

## 4. Conclusions

The venoms of snakes from several genera were tested for toxicity to protozoa *T. pyriformis*. The cobra venoms were found to possess the highest activity and *N. sumatrana* venom was the most active. The targeted isolation of toxic polypeptides resulted in purification of cytotoxins belonging to three-finger toxin family. The cytotoxins from other cobra venoms were active against *T. pyriformis* as well. Thus, using the ciliate *T. pyriformis* as an example, for the first time we have shown that cobra venom cytotoxins have antiprotozoal activity.

## 5. Materials and Methods

All salts obtained from local suppliers were of analytical grade or higher. Cytotoxins from *N. oxiana* venom were purified as described [29] and cytotoxin 3 and its glycosylated form were isolated from *N. kaouthia* venom as described [30]. Fluorescently labeled cytotoxin II from *N. oxiana* was prepared as described [34]. Acetonitrile was purchased from Kriochrom (Sankt Petersburg, Russia), and trifluoroacetic acid from Merck KGaA (Darmstadt, Germany).

### 5.1. Snake Venoms

Cobra (N. kaouthia, N. melanoleuca, N. naja, N. nigricollis, N. nivea, N. oxiana, N. sumatrana) and viper (Vipera nikolskii, V. ursinii, Macrovipera lebetina, Bitis arietans, Echis multisquamatus) venoms were obtained as described earlier [30,35]. The krait Bungarus fasciatus venom was obtained as described in [36]. Venoms collected were dried over anhydrous CaCl_2_ and stored at −20 °C until use. Venoms of Agkistrodon contortrix contortrix, Calloselasma rhodostoma and Gloydius blomhoffii were a kind gift of Professor T. Morita (Meiji Pharmaceutical University, Tokyo, Japan).

### 5.2. Anti-Protozoan Activity Measurements

Anti-protozoan activity was measured on the ciliate *T. pyriformis* using a BioLaT-3.2 instrument operating under the AutoCiliataXP program (Europolytest Ltd., Moscow, Russia) essentially as described [21]. *T. pyriformis* (strain WH14 from the collection of the Russian Research Institute of Veterinary Sanitary, Hygiene and Ecology, Moscow, Russia) was cultivated in the medium containing 0.5% pancreatic casein hydrolysate (Merck KGaA), 0.5% glucose, 0.1% Springer 0251 yeast extract (Bio Springer, Maisons-Alfort, France) and 0.1% NaCl. For the activity determination, the solution tested (5 to 50 μL) was added to the suspension of *T. pyriformis* cells in measuring wells of the instrument, and the number of survived cells was counted each minute using the AutoCiliataXP program for a period of up to 1 h. In brief, the wells were positioned under two video cameras and the survived cells were cyclically counted. The real time dependences of the number of survived cells on the time were shown on the display. Only number of moving cells at each minute was counted. At the end of experiment all the data were saved as an Excel file.

### 5.3. Venom Fractionation

Gel filtration was performed on a Superdex^TM^ 75 column (10 × 300 mm, Cytiva, Marlborough, MA, USA), equilibrated with 0.1 M ammonium acetate (pH = 6.2), at a flow rate of 0.5 mL/min. The eluting proteins were detected by absorbance at 280 nm. In total 63 mg of *Naja sumatrana* venom were fractionated. The fractions were pooled as shown by horizontal bars in Figure 2A, freeze-dried and analyzed for lethal activity against *T. pyriformis*. For this purpose, each freeze-dried fraction was dissolved in 500 µL of water, 120 µL of this solution was freeze-dried again to remove ammonium acetate and the dried residue was dissolved in 100 µL of water for toxicity measurement. From 1 to 10 µL of the solution was added to the measuring well of BioLaT-3.2 instrument containing 600–1000 infusorians in 300 µL of the medium and the number of surviving organisms was registered for up to 1 h. The faction IV (Figure 2A) toxic to *T. pyriformis* was separated by ion-exchange chromatography on a HEMA BIO 1000 CM column (8 × 250 mm, Tessek, Prague, Czech Republic) with a gradient of 5–700 mM ammonium acetate (pH 7.5) in 140 min, at a flow rate of 0.5 mL/min. Fractions obtained (Figure 2B) were further screened for lethal activity as described for gel-filtration fractions and toxic fractions 10 and 11 were further separated by reversed-phase chromatography on a Bio Wide Pore C18 column (10 × 250 mm, Merck KGaA) in a gradient of acetonitrile 20–50% in 60 min in the presence of 0.1% trifluoroacetic acid, at a flow rate of 2.0 mL/min.

### 5.4. Mass Spectrometry and Peptide Mass Fingerprinting

MALDI mass-spectrometry and peptide mass fingerprinting were performed as described [37].

### 5.5. Microscopy

The microscopy studies were performed using an IX-71 inverted microscope operating under the CellA program (Olympus Corporation, Tokyo, Japan). The suspension of *T. pyriformis* cells in the cultivating medium (40 µl) was placed to the Greiner flat bottom 96-well plate (Merck KGaA) and the solution of cytotoxin 2 *N. oxiana* or rhodamine labeled cytotoxin 2 from *N. oxiana* were applied to final concentration of 1 mg/mL. Images were captured at the certain time intervals at automatic exposure time in the visible light or in fluorescence mode using the U-MNG fluorescence cube (excitation at 530–550 nm). Fluorescent images were taken first.

## Figures and Tables

**Table 1:** Death times of 50% of the ciliates in the presence of cobra venoms.

Species	Venom Concentration, mg/mL	Time to Death 50% of Ciliates, min
*N. nigricollis*	0.33	1.75
*N. sumatrana*	0.33	2.5
*N. naja*	0.33	6.6
*N. kaouthia*	0.33	14.0
*N. oxiana*	1.61	2.5
*N. nivea*	1.61	9.6
*N. melanoleuca*	1.61	18.0

**Table 2:** Death time of 50% of ciliates in the presence of cobra cytotoxins. Toxin concentration is 0.95 mg/mL.

Toxin	Toxin Type	Time to Death 50% of Ciliates, min
CX2 *N. oxiana*	P-type	1.5
NSIV-11-3	P-type	2.0
CX3 *N. kaouthia*	S-type	2.3
CX1 *N. oxiana*	S-type	5.4
NSIV-10-1	S-type	17.5
NSIV-11-1	n.d.	8.5
NSIV-11-2	n.d.	5.8

**Table 3:** Comparison of theoretical digestion fragments of cytotoxin 2c and cardiotoxin 5A *N. sputatrix* with those found in tryptic digests of proteins Ns4-10-1 and Ns4-11-1 from *N. sumatrana*.

Molecular Masses, Da				Position in the Sequence	Amino Acid Sequence
Determined		Calculated			
NsIV-10-1	NsIV-11-1	Cytotoxin 2C	Cardiotoxin 5A		
576.15	576.13	576.28		13–18	TCPAGK
No signal	598.25	598.40		32–36	VPVKR
605.22	605.20	605.34		1–5	LKCNK
640.18	640.16	640.32		19–23	NLCYK
No signal	646.26	646.41		45–50	SSLLVK
834.27	834.22	834.38		37–44	GCIDVCPK
879.43	879.38	879.53		6–12	LVPLFYK
No signal	940.30	940.46		24–31	MYMVATPK
973.25	973.23	973.39		51–58	YVCCNDTR
1190.27	1190.23	1190.44		51–60	YVCCNDTRCN
1365.57	No signal		1365.73	24–35	MFMVSNLTVPVK
1521.65	No signal		1521.83	24–36	MFMVSNLTVPVKR
